# Decision-makers’ experiences with rapid evidence summaries to support real-time evidence informed decision-making in crises: a mixed methods study

**DOI:** 10.1186/s12913-023-09302-0

**Published:** 2023-03-25

**Authors:** Ahmad Firas Khalid, Jeremy M. Grimshaw, Nandana D. Parakh, Rana Charide, Faiza Rab, Salim Sohani

**Affiliations:** 1 Centre for Implementation Research, Canadian Institutes of Health Research Health System Impact Fellowship, Ottawa Hospital Research Institute, Ottawa, ON Canada; 2 Centre for Implementation Research, Ottawa Hospital Research Institute, Ottawa, ON Canada; 3 School of Global Health, York University, Toronto, ON Canada; 4 Health in Emergencies, Canadian Red Cross, Ottawa, ON Canada; 5 Faculty of Medicine, University of Ottawa, Ottawa, ON Canada; 6 Faculty of Health Sciences, McMaster University, Hamilton, ON Canada; 7 Department of Health Research Methods, Evidence and Impact, McMaster University, Hamilton, ON Canada

**Keywords:** Knowledge translation, Evidence-informed decision-making, Evidence summaries, Science communication, Evidence, Data

## Abstract

**Background:**

There is a clear need for research evidence to drive policymaking and emergency responses so that lives are saved and resources are not wasted. The need for evidence support for health and humanitarian crisis is even more pertinent because of the time and practical constraints that decision-makers in these settings face. To improve the use of research evidence in policy and practice, it is important to provide evidence resources tailored to the target audience. This study aims to gain real-world insights from decision-makers about how they use evidence summaries to inform real-time decision-making in crisis-settings, and to use our findings to improve the format of evidence summaries.

**Methods:**

This study used an explanatory sequential mixed method study design. First, we used a survey to identify the views and experiences of those who were directly involved in crisis response in different contexts, and who may or may not have used evidence summaries. Second, we used the insights generated from the survey to help inform qualitative interviews with decision-makers in crisis-settings to derive an in-depth understanding of how they use evidence summaries and their desired format for evidence summaries.

**Results:**

We interviewed 26 decision-makers working in health and humanitarian emergencies. The study identified challenges decision-makers face when trying to find and use research evidence in crises, including insufficient time and increased burden of responsibilities during crises, limited access to reliable internet connection, large volume of data not translated into user friendly summaries, and little information available on preparedness and response measures. Decision-makers preferred the following components in evidence summaries: title, target audience, presentation of key findings in an actionable checklist or infographic format, implementation considerations, assessment of the quality of evidence presented, citation and hyperlink to the full review, funding sources, language of full review, and other sources of information on the topic. Our study developed an evidence summary template with accompanying training material to inform real-time decision-making in crisis-settings.

**Conclusions:**

Our study provided a deeper understanding of the preferences of decision-makers working in health and humanitarian emergencies about the format of evidence summaries to enable real-time evidence informed decision-making.

**Supplementary Information:**

The online version contains supplementary material available at 10.1186/s12913-023-09302-0.

## Contributions to the literature

- Research has shown that the scientific evidence does not always have a large influence on decisions in health and humanitarian emergencies primarily because of the format it is presented that is not conducive for real-time decision-making in crises.
- The need for evidence support for health and humanitarian crisis is even more pertinent because of the time and practical constraints that decision-makers in these settings face.
- These findings contribute to recognized gaps in the literature and practice, including preferences of decision-makers working in health and humanitarian emergencies about the format of evidence summaries to enable real-time evidence informed decision-making.
- This is the first study to produce an evidence summary template conducive for evidence informed decision-making in health and humanitarian emergencies and an example of an application of the summary to a real-world health and humanitarian emergency.

## Background

Crises like the COVID-19 pandemic, climate related crisis (e.g., Pakistan floods) and the refugee crisis (e.g., Syrian, and Ukrainian refugees) are placing a strain on healthcare services, resources, and healthcare workers. The unprecedented scale of investment in strengthening health systems has led many stakeholders to demand the use of more reliable evidence for decision-making to ensure that desired impacts are achieved at acceptable costs [1–3]. There is a clear need for research evidence to drive policymaking and emergency responses so that more lives are saved, and resources are responsibly used. The need for evidence support for health and humanitarian crisis is even more pertinent because of the time and practical constraints that decision-makers in these settings face. To improve the use of research evidence in policy and practice, it is important to provide evidence resources tailored to the target audience [4]. One area to consider when seeking to strengthen the use of research evidence in crises is using rapid evidence summaries presented in a concise manner that can be easily understood by non-technical decision-makers in a short timeframe [5–16]. Little is known about health and humanitarian crises decision makers’ use of evidence resources. This study aims to explore decision-makers’ views of evidence summaries contributing further to our understanding of the impact of evidence summaries and the use of research evidence in decision-making in crises.

For the purpose of this study, we define research evidence as the output of research that has been conducted in a systematic way and reported in a transparent manner [1]. Our definition of research evidence includes evidence described in both empirical papers (e.g., observational studies, surveys, and case studies) and conceptual papers (e.g., theoretical papers). This includes primary and secondary research (e.g., systematic reviews and other forms of evidence synthesis). Research evidence may appear in indexed bibliographic databases or in grey literature. We distinguish such research evidence from other types of information, including tacit knowledge or ordinary knowledge [17] and stakeholder opinions.

For the purpose of this study, we define evidence summaries as a short summary of the best available evidence stemming from secondary research based on a defined question. To date, there is no agreed upon evidence summaries template to support decision-making in crises. There are several organizations that develop and disseminate evidence summaries curated for decision-making in crises. For example, Evidence Aid is a non-profit organization that specializes in collating and summarizing evidence about how to effectively prepare for and respond to disasters and emergencies [18]. There are other organizations that develop general evidence summaries (e.g., Cochrane). However, these summaries are not always specific to crisis settings making them suboptimal for use by decision-makers working in crisis settings.

Research evidence on the usefulness and effectiveness of evidence summaries to inform decision-making in crisis zones is lacking. The broader literature supports the use of rapid evidence summaries to inform decision-making in other settings [19–22]. A critical interpretive synthesis by Khalid et al. found that rapid evidence summaries can be useful in humanitarian aid sector given the need for evidence to be presented in a concise manner that can be easily understood by non-technical decision-makers in a short timeframe [5]. This study has two key objectives. First, to gain real-world insights from decision-makers working in health and humanitarian emergencies about how they use evidence summaries, their preferences, and impacts on decision-making; and second, to use our findings to improve the format of evidence summaries by identifying the most effective summary components for increasing decision-makers’ use of the evidence in health and humanitarian emergencies.

## Methods

### Design

We used a sequential mixed method study design. This type of design combines elements of qualitative and quantitative research approaches to ensure that we arrive at the breadth and depth of understanding on the use of evidence summaries to inform decision-making in crises. First, we used a survey to identify the views and experiences of evidence summaries by decision-makers who were directly involved in crisis response in different contexts. Second, we used the insights generated from the survey to help inform qualitative interviews with decision-makers in crisis zones to derive an in-depth understanding of how they use evidence summaries and the desired format of evidence summaries to gain real-time support in using evidence to inform decision-making. The reporting of this study followed the checklist for mixed methods research manuscription preparation and review [23].

### Sampling of participants

Decision-making processes are complex, especially when dealing with health and humanitarian emergencies where the need to make real-time decisions is imperative and often requires a network of stakeholders with different types of expertise. We purposively sampled participants who have used evidence summaries before, and those who have not. We aimed to survey and interview a diverse group of decision-makers from various countries working in different crises, with a wide range of experience working in health policy and management decisions. For inclusion, participants had to belong to one of the following five categories based on their roles in decision-making in crisis response and where appropriate, across the humanitarian aid, health system, and health research system sectors: 1) senior decision-makers’ (e.g., presidents, directors); 2) field managers (e.g., field coordinators, heads of missions) directly involved in coordination and management of crisis situations; 3) healthcare providers (e.g., doctors, nurses) involved with either the development of medical guidelines in crisis situations or directly delivering medical care to people in crisis situations; 4) advisors directly involved in advising about policy development and implementation strategies; or 5) analysts and researchers directly involved in responding to research evidence requests from the previous four categories of participants.

### Participant recruitment and sample size

A two-stage sampling approach was used to identify and recruit key informants [24, 25]. The first stage included identifying participants in the five categories of decision-makers. The second stage involved snowball sampling by which research participants in the first stage were asked to identify any additional informants. To capture users who have and have not used evidence summaries, we did the following: first, we sent email invitations to those listed on a publicly available contact list for a quality improvement exercise conducted at the Canadian Red Cross that focused on documentation and technical learning for Epidemic Prevention and Control efforts during the first wave of COVID-19 pandemic. Email reminders were sent out to those listed on the publicly available contact list. Second, we reached out to specific organizations that have been producing evidence summaries (e.g., Evidence Aid) to request that they share our invitations on their websites and social media platforms. Finally, the research team created a video to invite decision-makers to participate in the study. The video was shared on the research team twitter account. The video shared on public domain profiles assisted us in reaching out and recruiting those who are involved in crisis zones but are not part of the Evidence Summary websites.

We aimed to complete at least 5 survey respondents and semi-structured interviews for each type of participant category (i.e., decision-makers’, field managers, healthcare providers) for both evidence summary users and non-users, while recognizing that this estimate was dependent on the availability of appropriate participants.

### Data collection methods

#### Phase one: quantitative study

Participants completed an online survey administered using Lime Survey (survey instrument available as an e-appendix). The survey was in English language and consisted of four parts with the first part focusing on participants background and demographic characteristics, the second part related to questions that captured participants’ research experiences and familiarity with evidence summaries, the third part specific for evidence summaries users experiences and preferences, and lastly questions directed at non-evidence summary users reasons for not using evidence summary websites and preferences for evidence summaries and platform hosting summaries. We aimed to have the same preference questions for both users and non-users. However, non-users were not as familiar with evidence summary websites and therefore, we had to slightly adapt their preference questions, which limited our ability to compare some of the preferred features between the two groups.

#### Phase two: qualitative study

Following completion of the survey, interviews were conducted via Zoom and lasted approximately 30 min. Interviews were conducted by the PI (Khalid) who holds a PhD in Health Policy and has experience in conducting semi-structured interviews. Interviews were also conducted by other members of the research team (Parakh) who received training by the PI. Interviews were audio-recorded after receiving informed consent from the participant, audio recordings were transcribed verbatim, and the written transcriptions were used for data analysis. Potentially identifying information (e.g., name) was removed at the time of transcription. We conducted the interviews in English, which is the language all our participants preferred to conduct the interview in. A pilot interview with a research team member was conducted to refine the interview questions. The questions began by asking individuals about their roles and experiences related to their research evidence needs to support decision-making in crises. For example, questions were asked around what type of information influenced their decision-making process, what sources of research evidence they use, and their knowledge of evidence summaries. The interview then focused on specific questions related to evidence summaries and the platforms on which they are hosted. We also showed participants an example of an evidence summary retrieved from Evidence Aid website to solicit their feedback on it.

### Data analysis

A mixed methods approach to data analysis was employed. For the survey, quantitative data was summarized using simple descriptive statistics (numbers, percentages, frequencies, and cross tabulation). Data analysis included calculating descriptive statistics related to all assessed measures, including background of participants, their experiences with evidence summaries, and their attitudes towards using them. For the semi-structured interview data, we used a deductive framework analysis approach towards our collected data [26, 27]. Framework analysis is a qualitative method that can be applied to research that has specific questions, professional participants, and a limited time frame. This approach allowed us to describe and interpret what was happening in a particular setting (i.e., use of evidence summaries). It involves a five-step process: familiarization (i.e., immersing ourselves in collected data making notes of key ideas and recurrent themes), identifying a thematic framework (i.e., recognizing emerging themes), indexing (i.e., using NVivo to identify sections of data that correspond to particular themes), charting (i.e., arranging identified sections of data into table exhibits), and mapping and interpretation (i.e., analyzing key characteristics from the exhibits).

## Results

Our results section starts with a brief description of the key informants we surveyed and interviewed to arrive at a comprehensive understanding of the views and preferences of decision-makers with rapid evidence summaries to support real-time evidence informed decision-making in crisis settings. We then describe the preferences of evidence summary users and introduce an evidence summary template based on the insights gathered.

### Participants’ characteristics

We recruited 16 participants from our first stage of sampling, and 10 additional participants were identified through snowball sampling**.** Participants participating = Twenty-six. Table 1 presents the characteristics of the participants. Additional file contains more details on the demographic background of our participants (see Tables 1, 2, and 3 in Additional file 1).

**Table 1:** Profiles of participants

Type of participant	% (n) T = 26	Organizational affiliations	Organizational types	Evidence summary user	Sex	Country of employment	Field/Sector
Senior Decision-maker	(*n* = 7)	Canadian Red Cross (*n* = *2)* PAHO (*n* = 1) Lebanese Association of Knights of Malta (*n* = 1) Nashua New Hampshire’s Gate City (*n* = 1) Bean Voyage (*n* = 1) Providence Emergency Management Agency and Emergency Operations Center (*n* = *1)*	NGO (*n* = 3) UN Specialized Agency (*n* = 1) Governmental Agency (*n* = 1) Other (*n* = 2)	3 No 4 Yes	4 F 3 M	USA (*n* = 2) Canada (*n* = 1) Switzerland (*n* = 1) Costa Rica (*n* = 1) Lebanon (*n* = 1) South Sudan (*n* = 1)	Health (*n* = 3) Education (*n* = 1) Other (*n* = 3)
Advisor	(*n* = 6)	Albany Medical College (*n* = 1) Canadian Red Cross (*n* = 3) Pan American Health Organization (*n* = 1) International Committee of the Red Cross (*n* = 1)	Academic Institution (*n* = 1) Non-Profit Humanitarian Organization (*n* = 3) NGO (*n* = 1) UN Specialized Agency (*n* = 1)	4 No 2 Yes	2 F 4 M	Canada (*n* = 3) Lebanon (*n* = 1) PAHO Regions (*n* = 1) USA (*n* = 1)	Health (*n* = 5) Other (*n* = 1)
Researcher	(*n* = 6)	Canadian Red Cross (*n* = 3) Universidad Nacional de Colombia (*n* = 1) Instituto Nacional de Cancerología (*n* = 1) Unknown (*n* = 1)	NGO (*n* = 2) Governmental Agency (*n* = 2) Academic Institution (*n* = 1) Other (*n* = 1)	3 No 3 Yes	5 F 1 M	Canada (*n* = 2) Colombia (*n* = 2) Ethiopia (*n* = 1) LMIC (*n* = 1)	Health (*n* = 5) WASH (*n* = 1)
Program Manager	(*n* = 5)	Canadian Red Cross (*n* = 3) Pan American Health Organization (*n* = 1) Norwegian Refugee Council (*n* = 1)	NGO (*n* = 3) Other (*n* = 2)	3 No 2 Yes	1 F 4 M	USA (*n* = 1) Honduras (*n* = 1) Nepal (*n* = 1) Bangladesh (*n* = 1) South Sudan (*n* = 1)	Health (*n* = 2) WASH (*n* = 1) Livelihoods (*n* = 1) Other (*n* = 1)
Field manager	(*n* = 1)	International Committee of the Red Cross (*n* = 1)	Other (*n* = 1)	1 Yes	1 F	South Sudan (*n* = 1)	Protection (*n* = 1)
Healthcare Provider	(*n* = 1)	Unknown (*n* = 1)	Other (*n* = 1)	1 No	1 F	Canada (*n* = 1)	Health (*n* = 1)

### Quantitative results

#### Preferences of evidence summaries users

Table 2 presents summary of findings. Ten participants had previous experience of using evidence summaries. The top three reported sources used for evidence summaries were Cochrane (67%), Evidence Aid (58%), and Relief Web (58%). The majority of users reported that they occasionally referred to evidence summaries (around two to four times per month). When asked about how information found in evidence summaries influenced participants’ decision-making, only 25% reported it as having a “great” influence and 75% reported that this information “somewhat” influenced their decisions. It is important to highlight here that 41% of participants indicated that they relied on information from evidence summaries solely to make a COVID-19 health decision. Interestingly, 67% of participants reported that this information was “somewhat informative” and 42% gave a “low” rating for having these summaries available on time. The most preferred presentation of these summaries was a written summary (50%) and a combination between infographics, figures, and written summaries (33%). Finally, the top five preferred features selected to be included in an evidence summary were: “*Concise summary of the evidence, including benefits, harms and costs, and implementation considerations and recommendations”, “A link that directs you to the full text source”*, *“Full text pdf option”, “Assessment of the quality of the evidence”, and “date of search strategy (i.e., how up-to-date is the evidence)”.*

**Table 2:** Summary of findings table

Preferences of evidence summary users’		N (%)
Sources most familiar with to access evidence summaries^a^	*Cochrane*	8 (66.6)
	*Evidence Aid*	7 (58.3)
	*Relief Web*	7 (58.3)
	*Campbell Collaboration*	4 (33.3)
	*International Initiative for Impact Evaluation (3ie)*	4 (33.3)
	*Victoria’s Hub for Health Services and Business (health.vic.gov.au)*	2 (16.6)
	*Africa Center for Evidence (University of Johannesburg)*	0 (0)
	*Other*	0 (0)
Frequency of referring to evidence summaries during the COVID-19 pandemic^a^	*Very frequently (more than 7 times)*	3 (25)
	*Frequently (4 to 6 times)*	2 (16.6)
	*Occasionally (2 to 4 times)*	7 (58.3)
	*Rarely (less than 2 times)*	0 (0)
Extent information in the evidence summaries influenced final decisions	*To a great extent*	3 (25)
	*Somewhat*	9 (75)
	*Very little*	0 (0)
	*Not at all*	0 (0)
Challenges with finding and using research evidence in crises^b^	*Websites require moving through several webpages to reach the desired information*	6 (50)
	*Lack of evidence relating to specific crisis areas*	4 (33.3)
	*Lack of evidence relating to my field of work*	2 (16.6)
	*Some websites’ interface is not user-friendly*	1 (8.3)
	*Other*	2 (16.6)
Features would like to see within an evidence summary (if they were to use them)^a^	*Concise summary of the evidence, including benefits, harms and costs, and implementation considerations and recommendations*	11 (78.5)
	*Key messages in bullet point format*	9 (64.2)
	*Information about the research methods of the summarized systematic review*	8 (57.1)
	*Assessment of the quality of the evidence*	8 (57.1)
	*Infographics*	0 (0)
	*Indirectness assessment*	3 (21.4)
	*Date of search strategy (how up to date is the evidence)*	7 (50)
	*A link that directs you to the full text source*	13 (92.8)
	*Full text pdf option*	6 (42.8)
	*Full-text Citations*	3 (21.4)
	*Other*	2 (14.2)
	*Concise summary of the evidence, including benefits, harms and costs, and implementation considerations and recommendations*	0 (0)
Preferences of non-evidence summary users’		N (%)
Reasons for not using Evidence Summaries Websites	*No specific reason*	4 (28.5)
	*Never heard of them before*	3 (21.4)
	*I do not usually need such tools for my work*	1 (7.1)
	*I find such kind of evidence as “very little informing”*	0 (0)
	*I prefer reading the in-depth evidence for a clearer picture*	2 (14.2)
	*Other*	4 (28.5)
Features to help them decide which evidence summary website to choose	*Based on credibility of website*	9 (64.2)
	*Based on the number of years of website’s activity*	1 (7.1)
	*Would rely on a colleague’s previous experience*	3 (21.4)
	*Other*	1 (7.1)
Extent they anticipate evidence summaries will have influence on their future decision making	*To a great extent*	4 (28.5)
	*To a moderate extent*	8 (57.1)
	*To a small extent*	2 (14.2)
	*Not at all*	0 (0)
Anticipate using evidence summaries in the future	*Yes*	9 (64.2)
	*Maybe*	5 (35.7)
	*No*	0 (0)
Features would like to see within an evidence summary (if they were to use them)^a^	*Concise summary of the evidence*	11 (78.5)
	*Information about the research methods of the summarized research paper*	9 (64.2)
	*Assessment of the quality of the evidence*	8 (57.1)
	*Infographics*	8 (57.1)
	*Risk of bias assessment*	0 (0)
	*Indirectness assessment*	3 (21.4)
	*Date of search strategy*	7 (50)
	*A link that directs you to the full text source*	13 (92.8)
	*Full text pdf option*	6 (42.8)
	*Implication’s considerations including contextual factors*	3 (21.4)
	*Equity related considerations*	2 (14.2)
	*Other*	0 (0)

^a^Half of the participants were engaged in COVID-19 response 6/12)

^b^Can select more than one answer (total is not 100%)

#### Preferences of non-evidence summary users

Fourteen participants had no prior experience of using evidence summaries. They either did not have a specific reason for not using evidence summaries or had never heard about them before. Non-users indicated the following preferred features within an evidence summary: *“Having access to full text”, “Having a concise summary”, “Including information about the research methods”, “Having infographics”, and “Including quality assessment of the evidence”*. When asked about features that would help them choose which evidence summary website to refer to, the majority reported credibility as the main factor (64%). Finally, the majority of non-users reported that they anticipate using evidence summaries in the future (64%), and more than half of them (57%) stated that information from these summaries might moderately influence their future decision making.

## Qualitative results

### Participants preferred sources for information in crises

Two key themes emerged from our interviews on preferred sources for obtaining information in a crisis. First, there is a reliance on internal sources (e.g., in-house technical advisors & colleagues) to obtain relevant information. Decision-makers we interviewed discussed the benefits of having in-house technical advisors who can retrieve contextually relevant information stating:

“*It’s not easy to access information in the midst of a crisis, you have a lot of research, I don’t want to wait long so I use my technical advisor within the technical team that are based in my country level who then can dig into the information and make sense of what might be useful for me.” – Evidence summary user 1*

Second, Google and Google Scholar were repeatedly cited as a key source for obtaining research evidence in crises. A key informant stated that the reasons behind frequently using Google and Google Scholar is because they are:

“*When you need evidence and information right away, Google can give you that immediately. Journal articles don’t provide that type of information right away”– Evidence summary user 2*

### Challenges with finding and using research evidence in crises

Our interviews with participants provided us with additional challenges participants face in finding and using research evidence in crises. First, the lack of time and increased burden of responsibilities during crises creates a challenging environment to find and use relevant evidence to inform decision-making. A key stakeholder working in crisis settings at a non-governmental agency highlighted how the lack of time to find information makes it imperative to find:

“*Filtered information on a trustworthy site*” *Non-evidence summary user 1*

Second, there are challenges with having access to reliable internet connection that would enable download of large documents with one decision-maker stating:

“*In crises, there are often internet connectivity download issues especially with large documents. Halfway through browsing, everything can stop, and you don’t have the bandwidth, or the quality of connection is reduced which means you have to redo the search all over again.” – Evidence summary user 1*

Third, challenges arise in regard to the large volume of available data that is not analyzed and translated into user friendly summaries. For example, a decision-maker stated the following:

*“We have a lot of data points. Technical units collect massive amounts of data that includes numbers on people, numbers on meetings, numbers on sites. What they haven’t been able to do is to partner with say academic to do analysis of the data and to conduct impact studies.”– Non-evidence summary user 2*

Lastly, decision-makers working in organizations that carry out field operations in crises highlighted that research evidence, as defined in this study, is not considered a main source of information for operational teams. Instead, lessons learned and tacit knowledge are the main sources of information:

“*Most of the people who are in the field working in crises do not rely on scientific and evidence-based data in their work, instead they rely on lessons learned and what they have seen in their careers.*” *– Evidence summary user 3*

### Challenges with finding and using research evidence during COVID-19 pandemic

Participants noted that during the COVID-19 pandemic, there was a plethora of different websites on the same topic making it challenging to find and use evidence in real-time to inform decision-making. One decision-maker stated:

“*Before COVID-19 I would go to the CDC or FEMA website and was able to find one single source of evidence on there but during COVID-19 I would go on the same site and there would be 20 different web pages about the same topic.*” – *Evidence summary user 3*

Decision-makers also noted that finding and using research evidence to inform decision-making during the COVID-19 pandemic was a slow and tedious process given the rapidly emerging evidence, with one decision-maker stating:

“*There is a difference between finding and using evidence pre-pandemic and post-COVID-19 pandemic. During the pandemic everything was slow, and it was hard to find the relevant evidence because it was either not available or rapidly changing*” *– Evidence summary user 4*

A senior decision-maker working in emergency preparedness and response at a government agency also noted that during the COVID-19 pandemic, there was little information available on evidence websites specifically around preparedness and response measures. Most of the available evidence was focused on clinical interventions and clinical pathways of the virus. As stated by that decision-maker:

“*A lot of the online resources for evidence were much more health-specific focused, and less so on preparedness and mitigation efforts. What we really needed and couldn’t find much of is evidence around the protective actions we need to take, the types of policies we should implement, and what are the population level impacts from the virus and less so on clinical interventions and symptomatology of the virus*” *– Evidence summary user 4*

### Participant’s preferences of online websites hosting evidence summaries

Several features of online websites hosting evidence summaries were cited by participants as important facilitators of real-time evidence use in crises. First, the credibility of the evidence summary website is a main factor in deciding whether to use the site to find and use research evidence summaries. This is partly because of the lack of time to navigate a new site, but also because of concerns around the quality of evidence presented. A participant we interviewed highlighted this by stating:

*“If you’re reading something on Cochrane or Evidence-Aid website, you would expect that is something of good quality and that you can trust the evidence presented” – Evidence summary user 5*

Second, the ability to access online websites of evidence summaries free of charge is a key facilitator with one decision-maker stating:

“*If it is not free of charge, then it is not useful. The whole objective is to make sure people can access evidence on a timely basis to inform their decision-making, so if you are putting a restriction of having to pay to access the evidence summary then it is defying the objectives of ensuring timely evidence informed practice*” *– Non-evidence summary user 3*

Third, the ability to search by topic is considered a useful feature of online websites. Participants noted the need to categorize findings according to crisis types, population, or an intuitive crisis relevant framework. One of the challenges noted by decision-makers on categorizing summaries based on topics is “*figuring out which topic my question that I need answers for falls under*”. Advanced filter features that allow decision-makers to filter the searches by region, crisis type, phase of emergency response, different disaster response sectors, etc., are a useful tool noted by decision-makers.

“*A clearly searchable scheme where we can search by key terms is important. For example, being able to search by phase of emergency management, preparedness or mitigation versus response and recovery, entities involved in the response whether they are more transportation-related, health-related or school-related.” – Evidence summary user 3*

Fourth, the linguistic accessibility for the online website and the summaries was cited as important. A decision-maker emphasized the need for the availability of multiple language summaries stating:

“*One of the most important things that I care about is language availability. This is because of the contexts and regions we work in. If I have to inform the Minister of Health from one of our member states, then the summary needs to be in the language of that member state*” *– Evidence summary user 5*

Fifth, participants prefer online websites that follow web accessibility norms and regulations. Web accessibility means that websites providing evidence summaries are designed and developed so that people with disabilities can use them. This means that people can perceive, understand, navigate, and interact with the Web. A decision-maker noted Evidence Aid’s web accessibility gradually improved gradually over time:

“*Evidence Aid improved a lot in terms of accessibility. The site is now simpler with less information and better organization. This makes it more in line with the norms and regulations of Web accessibility. However, I want to emphasize the need for platforms to be more inclusive in terms of accessibility standards. It’s not an expensive task, it just requires continued support from the organization*” *– Evidence summary user 5*

Sixth, decision-makers prefer online websites that clearly explain on the homepage what the site is focused on and provide clear instructions on how to navigate the site to arrive at evidence summaries. A participant highlighted this by stating:

“*Given that we work in crises, we do not have the time to go and conduct research and look deeper for proper sources so having access to websites where it is clear what the focus of the site is and having access to reliable information on the site that is easily accessible is very helpful” – Non-evidence summary user 3*

Lastly, researchers responding to the knowledge needs of decision-makers noted a preference for an alert function on online platforms where they can receive email notifications if there is new research or newly updated evidence summary with one researcher stating:

“*Subscription option is an important feature where we can receive notification by email if there is any new research or new update available on the evidence especially during the COVID-19 pandemic” – Non-evidence summary user 4*

## Participant’s preferences and views of using evidence summaries

Overall, our participants view evidence summaries as helpful tools to inform real-time decision-making with one decision maker stating:

*“When I need that quick review of the current evidence then evidence summaries are awesome – they are great”– Evidence summary user 6*

Some non-evidence summary users noted that part of the reason they are not using evidence summaries is that they did not know they exist. A decision-maker we interviewed highlighted that organizations that are producing evidence summaries should:

“*Publicize that they produce evidence summaries, and they should engage with humanitarian organizations and actors through a communication strategy to disseminate the evidence summaries*”*– Non-evidence summary user 3*

## Participant’s preferences and views of evidence summaries

A consistent theme identified was that evidence summaries should be presented in a clear, succinct, and action-oriented format (e.g., bullet points and actionable checklists). A senior decision-maker highlighted this by stating:

“*In my role (as a senior decision-maker), I am looking for a clear answer. I work in emergency response, and I want the evidence presented in clear bullet points.*” *– Non-evidence summary user 5*

Decision-makers also noted the need for evidence summaries to be written in simple, jargon free language with a decision-maker stating:

“*The simpler the evidence summaries are, the easier it will be to digest.”*– *Evidence summary user 1*

## Participant’s preferences and views of evidence summaries during COVID-19 pandemic

Decision-makers noted a preference for presenting the chronological timeline of changes in the evidence with one decision-maker indicating:

“*With a novel incident like COVID-19 the evidence was fast changing and as the incident (COVID-19 pandemic) progressed along, there were changes. For example, the evidence around face masks was changing in real-time and we did not know whether we should be promoting its use in the community. A chronological timeline of how evidence is changing over a period of time helps us to tell a story as to why we chose which interventions at what time, and so we can really measure whether our strategies are working or not, but it also ensures that we know what the most current evidence is because it’s otherwise tough to sort through and sift through it all”– Evidence summary user 3*

Decision-makers also indicated a preference for the date of search strategy clearly highlighted. This was a particularly prominent finding when interviewing decision-makers that worked in COVID-19 pandemic response stating:

“*For COVID-19 pandemic, the evidence was quickly changing, and I needed to know from the evidence summary if the information was the most up-to-date to make sure that the answer presented in the summary is still valid for today*” - *Non-evidence summary user 6*

## Evidence summary template

**Fig. 1:**
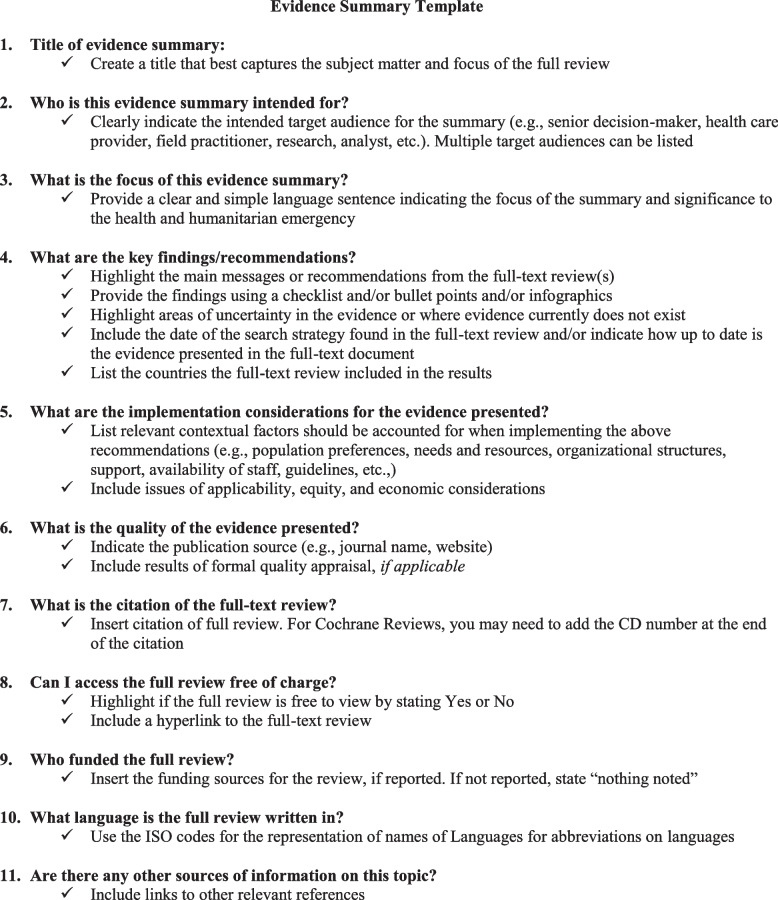
Evidence summary template (see Figure file)

Figure 1 provides a template of an evidence summary based on the views and preferences of decision-makers working in crisis zones. Evidence summary components are presented in the order most preferred by our key informants. We elaborate further on the key components of the evidence summary with supporting statements from our key informant interviews below. An example of an application of the evidence summary template to an evidence summary request received at the Canadian Red Cross is provided in additional file (see Supplementary file 1: Fig. 1). The example has been adapted to fit organizational context and decision-maker needs.

### Target audience

Identifying the target audience for the evidence summaries was cited by key informants as an important element when drafting summaries. One researcher with experience in creating evidence summaries stated:

*“It is very important to know who the target audience for the evidence summary because it differs if the summary is written for researchers as opposed to field staff working in operations who wants the evidence presented in a specific format.”– Evidence summary user 2*

### Issue statement

Decision-makers noted the preference for a one-line sentence at the beginning of the evidence summary that provides a quick, clear, simple plain language indication of what the summary is based on with one decision-maker stating:

“*You need that little, short sentence that tells you how this summary applies to you so that you can decide whether you want to go to the key findings or not. Something easy to understand for a practitioner*”*– Non-evidence summary user 7*

### Key findings

In addition to presenting the key findings from the full review, some decision-makers noted the need for background information related to the specific crisis. When we showed participants an example of an evidence summary retrieved from Evidence Aid website to solicit their feedback on it, they were not in favor of a text heavy summary. Instead, participants indicated their preference for key findings to be presented in a check list, bullet points, graph, or infographic format with one decision-maker stating:

*“Can the key findings be presented in a graph or in an infographic instead of it being in word text format because it will be better visually and easier to digest”– Evidence summary user 1*

Some decision-makers preferred to have key findings from the full review presented in infographics: stated in an interview preference for infographics to present the key findings from the full review:

“*The infographic needs to capture the key findings from the research and include the key recommendations to me as a decision-maker. Those are the most critical information to go in the infographic”– Evidence summary user 1*

A decision-maker noted that an additional benefit to using infographics is that they can be easily converted into a social media post to disseminate knowledge to the public stating:

“*In the case of emergency management, time really matters, and infographics can get us (decision-makers) evidence in a quick format. They are also great because we can convert them into something that can be then consumed by the public. We can push them out through our social media or a press conference because they are easily consumed by the public*”*– Evidence summary user 4*

Decision-makers also noted the importance of highlighting areas of uncertainty in the evidence or where evidence currently does not exist. A decision-maker stated:

“*I would like to see a section in the summary about what we don’t know about it in terms of evidence or where there is no evidence available yet so that when somebody asks me the question of whether we (decision-makers) should be concerned about this? I can respond to them and say that there’s currently no evidence that shows that for example COVID-19 can be transmitted on surfaces*”*– Evidence summary user 4*

The preference to indicate the research methods (i.e., type of study design, countries included, etc.) from the full review as part of the findings section of the evidence summary yielded mixed results. Some decision-makers noted that:

“*Research methods are only important to researchers but when it comes to translating the evidence to action, it is not as relevant*”*– Evidence summary user 2* while others noted “*research methods provide an indication of how valid and reliable the results are and that’s why it is important to me*” - *Evidence summary user 7*

A decision-maker we interviewed that worked directly in COVID-19 response highlighted:

“*Most of my colleagues probably wouldn’t even understand the research methods employed by the full review because they do not have that kind of training, so it is a challenge to actually get some value out of that*”*– Evidence summary user 3*

### Implementation considerations

Participants noted the need for implementation considerations to be explicitly stated and directly linked to the key findings presented with a decision-maker stating:

*“I need to know the fact (i.e., findings) but I also want the implementation considerations highlighted so that if I need to take immediate action, I can do that. For example, with COVID-19 we needed not only needed to know the key actions to take in terms of social distancing, face masks and washing hands but we also needed to know how to implement such interventions to determine if it is feasible or not”– Evidence summary user 1*

Contextual implementation consideration emerged as a key theme in all our interviews. Decision-makers repeatedly noted the importance of having an explicit section in the evidence summary that highlights specific contextual information that decision-makers need to keep in mind when making decisions. A suggestion proposed by a decision-maker to capture contextual elements in the evidence summary is to include the list of countries or region that the evidence summary is focused on. Participants highlighted this by stating:

*“Contextual factors matter to me because when I am in the middle of project implementation in the field or designing for new projects, I need to know what contextual factors I need to keep in mind otherwise my projects will not be successful. I need to know if the evidence presented is applicable in low- and middle- income countries”– Non-evidence summary user 8*

### Assessment of the quality of evidence presented

Decision-makers noted the importance of having a neutral entity assessing the quality of the evidence presented with one decision-maker stating:

“*Assessment of the quality of evidence is very useful, especially if you wanted to use it for programmatic development or as justification for what you want to do”– Evidence summary user 1*

Another decision-maker noted that because time is limited when operating in crises zones, they “*do not have the time to validate the sources, and this is why I prefer summaries that assess the quality of evidence presented*” *– Non-evidence summary user* 9.

### Citation of full review

Decision-makers highlighted that having the full citation of the systematic reviews and other forms of evidence synthesis allows them to assess whether the source is one they can trust but gave it little preference in terms of order of appearance in the summary, with one decision-maker noting:

“*It is important to know where the sources are coming from and this is why citation is key, we want to see if this is a legitimate research study coming from a credible site*”– *Evidence summary user 4*

### Availability of the full review

Access to the full review the summary was written from was noted as an important feature in evidence summaries. A senior decision-maker noted that sometimes to access the full review:

“*A request has to be sent to the Librarian at the organization which could take three to four days for a response and the reality is that in some countries that we operate in a Librarian is not available to help us access full reviews”– Non-evidence summary user 10*

### Hyperlinks for the full review and further information on the topic

Decision-makers highlighted the need to have the full review hyperlinked within the evidence summary for easier access. They also noted the need for hyperlinks to additional information on the topic to be inserted at the end of the evidence summary along with tagging of key words. Decision-makers noted that the desire to access the full review or further information on the topic is dependent on what phase of the emergency response they are in with one decision-maker stating:

“*As a practitioner working in the field, I am probably not going to pull the full review or look for additional information as opposed to someone working in preparedness or mitigation efforts where they might have more time and are not operating in a time-sensitive context. The full review and additional information are more for planning purposes and not in the acute phases of disaster response*”*– Evidence summary user 3*

### Funding sources

Indicating the funding source behind the full-review was noted as a preference by decision-makers to determine if there are any conflict of interests present in the findings presented in the evidence summary with one decision-maker noting:

“*I would want to see where the authors received funding to conduct the research to determine credibility of the evidence presented and if there are any obvious conflicts of interests*”*– Non-evidence summary user 11*

### Page and word limit

Decision-makers consistently stated that they preferred of having a one-page summary of the evidence with a senior decision-maker highlighting this in their remarks:

“*I think the shorter, the better, especially when we are talking about non-researchers who want the evidence in the most succinct way possible. Half a page will be enough but definitely not more than one-page*”*– 6 Evidence summary user*

## Discussion

Our study provided a further information of the preferences of decision-makers working in health and humanitarian crises for the format of evidence summaries to enable real-time evidence informed decision-making. In situations where time or resources are limited, evidence summaries take precedence in informing decision-making over extensive systematic reviews. Short summaries with key actionable messages highlighted in a bullet point format or infographics are strongly preferred. The use of infographics and graphs are strongly preferred to visually illustrate the findings. Infographics are an effective way to present complex and rapidly available information in a visually appealing format and is considered directly useful for decision-making purposes [3, 4]. They serve as an important role in bridging the gap between evidence synthesis and evidence uptake. In addition, summaries that demonstrate a critical appraisal of evidence and are easy to use are strongly associated with increased use in crises.

This study emphasizes that decision-makers working in crises prefer curated information sources (e.g., in-house technical advisors and colleagues) and to access websites that are easy to use (e.g., Google and Google Scholar). The reliance on those two specific sources of information is a result of the time-pressured situations decision-makers working in crises are under. They are expected to make decisions in a short time frame, often with limited information to inform their decisions. In-house technical advisors can provide evidence to decision-makers in a format they can easily understand, trust, and act upon in real-time. The preference for contextualized evidence summaries also emerged as a key finding. This is understandable given that contextual factors may have an impact on the health equity of an intervention, the feasibility of implementing an intervention, and the acceptability of an intervention [28]. Including a contextual lens in evidence summary synthesis is critical for decision-makers working in health and humanitarian emergencies.

The study identified the following challenges decision-makers face when trying to find and use research evidence in crises: insufficient time and increased burden of responsibilities during crises, limited access to reliable internet connection, burden of navigating websites, a large volume of data not translated into user friendly summaries, a plethora of different websites on the same topic, and little information available on preparedness and response measures. These different challenges provide insight into potential areas of improvement that knowledge producers working at addressing the knowledge needs of decision-makers working in crises can consider. Stakeholders working in health and humanitarian emergencies have specific knowledge needs and these findings reaffirm the importance of conducting further scholarly work that focuses on better understanding on how to best support evidence use in crises.

### Findings in relation to other studies

Our findings that evidence summaries for ‘real-time’ evidence informed decision-making in crises are likely easier to understand than complete systematic reviews align with other published studies that examined the effectiveness of evidence summaries on health policymakers and health systems managers [5, 29–31]. Ensuring that contextual factors are presented in evidence summaries aligned with other studies that explored the considerable impact of presenting contextual factors on the willingness of decision makers to consider a recommended option [21, 28, 32–34]. Finally, this study complements existing literature in being the first study to specifically focus on evidence summary use by diverse stakeholders working in health and humanitarian emergencies, elaborating on the knowledge needs and challenges stakeholders face in finding and using evidence in crises, and putting forward an evidence summary template that aligns with the knowledge needs of decision-makers working in crises zones [19, 20, 22, 29, 35–37].

### Strengths and limitations

There are three strengths to this study. This is the first study to address the knowledge gap in our understanding of decision-making experiences with rapid evidence summaries to support real-time evidence informed decision-making in crises. Second, this study was strengthened by our mixed-method approach of incorporating surveys and interviews. Survey results gave an overview of attitudes and preferences of decision-makers using evidence summaries. The interviews illuminated survey responses by allowing us to further explore preferences and issues of participants. Overall, this approach enhanced the format of evidence summaries to support ‘real-time’ use of evidence in decision-making in crises. Third, we interviewed diverse types of stakeholders working in health and humanitarian emergencies encompassing various organizational affiliations to arrive at a comprehensive understanding of the preferred evidence summary template conducive for real-time evidence informed decision-making.

One challenge to this study is that although we aimed to have the same preference questions for both users and non-users of evidence summaries, non-users were not as familiar with evidence summary websites which required us to slightly adapt their preferences questions thereby limiting our ability to compare some of the preferred features between the two groups. To address this challenge, we presented evidence summary websites to non-users during our follow up interviews to gather their preferred features.

### Implications for practice

Our findings suggest six ways to improve the synthesis of evidence summaries to allow real-time evidence informed decision-making in crises:

1. Writing summaries in a clear, succinct, and action-oriented format (e.g., bullet points and actionable checklists).
2. Identifying the target audience the summary is intended for.
3. Highlighting the most up-to-date evidence on fast emerging crises where evidence might be lacking and/or is continuously undergoing updating (e.g., COVID-19 pandemic).
4. Standardizing the format of evidence summaries to make it easier for them to read it quickly in real-time.
5. Presenting the contents of evidence summaries in alternate formats (e.g., infographics, pictures and graphs, audio podcasts, videos, etc.). This will help in addressing the time limitation decision-makers face in the midst of a health and humanitarian emergency.
6. Emphasizing the role of organizational stewardship in ensuring that decision-makers have free access to evidence summaries but also dedicated time to read them to inform their decision-making.

The results of our study carry with them some implications in health and humanitarian emergency response. With the right knowledge management systems in place; our evidence summary template can streamline the process of responding to decision-makers knowledge needs during a crisis. In addition, training workshops geared at researchers tasked with synthesizing evidence summaries will be necessary to ensure understanding and compliance with the evidence summary template. Lastly, organizations tasked with synthesizing evidence summaries related to health and humanitarian emergencies can use our practical recommendations to improve the structure of evidence summary websites and summaries. They could also continue to solicit feedback from the users of evidence summaries to ensure that evidence websites and summaries best meet their knowledge needs.

### Future research

The next steps in research could be for researchers to explore stakeholders’ experience with using our proposed evidence summary template to test if this format has improved the use of research evidence in ‘real-time’ evidence informed practice. Additionally, researchers could conduct a user-testing study to evaluate stakeholders experience with using infographics to inform decision-making. Specifically, they can explore the format and style of infographics preferred by decision-maker working in health and humanitarian emergencies.

## Conclusions

Overall, our participants view plain language rapid evidence summaries as a helpful tool to inform real-time decision-making in crises. This study summarized the key elements stakeholders working in health and humanitarian emergencies prefer in evidence summaries and provided an evidence summary template that can be used by organizations or individuals tasked with synthesizing evidence summaries to inform decision-making.

## Supplementary Information

**Additional file 1:** **Figure 1. **Example of an application of the evidence summary template to a real-world crisis. **Table 1. **Demographics/ Participants’background. **Table 2. **Preferences for Users. **Table 3. **Preferences for Non-Users.

## Data Availability

All data generated or analysed during this study are included in this published article (and its supplementary information files).

## Abbreviations

LMIC: Low- and middle-income countries
NGO: Non-governmental organization
PAHO: Pan American Health Organization
WASH: Water, sanitation, and hygiene
