# Supplementary material for: Decision-makers’ experiences with rapid evidence summaries to support real-time evidence informed decision-making in crises: a mixed methods study
Source: BMC Health Serv Res. 2023 Mar 25;23:282. doi: 10.1186/s12913-023-09302-0 (PMC10039327; doi:10.1186/s12913-023-09302-0)
Supplement: Supplementary file 1 — Additional file 1: Figure 1. Example of an application of the evidence summary template to a real-world crisis. Table 1. Demographics/ Participants’background. Table 2. Preferences for Users. Table 3. Preferences for Non-Users. [file 12913_2023_9302_MOESM1_ESM.docx]

**Supplementary files:**

**Figure 1.** Example of an application of the evidence summary template to a real-world crisis

**Title:** Vaccine Mandate in the Pandemic to Endemic Transition Phase of COVID-19

**Target Audience:** Occupational Health and Safety (OHS), Senior Management, and Leadership of CRC

**Focus of Summary:** Research evidence on whether a 2-dose vaccine mandate for CRC staff provides sufficient protection during the pandemic to endemic transition phase.

**Key Recommendations and Findings:**

- Continue to mandate the 2-dose vaccine for CRC staff because a minimum of two mRNA doses provides high level of protection against severe health outcomes (e.g., hospitalization, death).

**
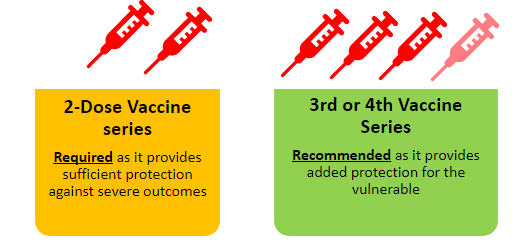
**

- With 2 doses of an approved mRNA vaccine, immunity against mild disease wanes after 6 months, but protection against severe COVID-19 illness remained high. [[2](https://bmcinfectdis.biomedcentral.com/articles/10.1186/s12879-022-07418-y),[3](https://www.thelancet.com/journals/lancet/article/PIIS0140-6736(22)00152-0/fulltext)]
  - 20-30% decline observed by 6 months after 2 doses against mild disease. [[2](https://bmcinfectdis.biomedcentral.com/articles/10.1186/s12879-022-07418-y),[3](https://www.thelancet.com/journals/lancet/article/PIIS0140-6736(22)00152-0/fulltext)]
  - Vaccine effectiveness remains greater than 70% against severity after 6 months. [[2](https://bmcinfectdis.biomedcentral.com/articles/10.1186/s12879-022-07418-y),[3](https://www.thelancet.com/journals/lancet/article/PIIS0140-6736(22)00152-0/fulltext)]
- Staff are recommended to stay up to date with their vaccines according to the latest PHAC guidelines for 3^rd^ and 4^th^ doses.
- Evidence on the duration of long-term protection following 3^rd^ and 4^th^ doses is limited.
  - The 3^rd^ dose effective to prevent Omicron infection has been documented at between 16-87 %; and between 66.7-90% at preventing severe disease outcomes up to 90 days after vaccination. [[4](https://www.who.int/news/item/17-05-2022-interim-statement-on-the-use-of-additional-booster-doses-of-emergency-use-listed-mrna-vaccines-against-covid-19),[5](https://www.mcmasterforum.org/docs/default-source/product-documents/living-evidence-syntheses/covid-19-living-evidence-synthesis-6.38---what-is-the-efficacy-and-effectiveness-of-available-covid-19-vaccines-in-general-and-specifically-for-variants-of-concern.pdf?sfvrsn=be5dca50_5)]
  - Early studies of the 4^th^ dose show some short-term benefit of an additional booster dose of mRNA vaccine in health workers, those over 60 years of age, or with immunocompromising conditions. [[4](https://www.who.int/news/item/17-05-2022-interim-statement-on-the-use-of-additional-booster-doses-of-emergency-use-listed-mrna-vaccines-against-covid-19),[5](https://www.mcmasterforum.org/docs/default-source/product-documents/living-evidence-syntheses/covid-19-living-evidence-synthesis-6.38---what-is-the-efficacy-and-effectiveness-of-available-covid-19-vaccines-in-general-and-specifically-for-variants-of-concern.pdf?sfvrsn=be5dca50_5)]
- The quality of evidence varies as this is an ongoing novel research area.

**Implementation Considerations:**

- Transition from using the term ‘fully vaccinated’ to ‘stay up to date on your vaccine doses’ in messaging to CRC staff.
- Despite removing the vaccine mandates for all federal employees, the Federal Government continues to strongly recommend that staff stay up to date with their vaccines. [[1](https://www.canada.ca/en/treasury-board-secretariat/news/2022/06/backgrounder-government-of-canada-suspends-mandatory-vaccination-for-federal-employees.html)]
- Decisions to remove or keep the vaccine mandates for CRC staff require special consideration of CRC's operational reality (high risk). These situations can likely exacerbate risks of severe disease outcomes to the people we serve.

**References and Further Information**

| Reference List | Source/Type | Open access | Funding source | Formal quality appraisal |
| --- | --- | --- | --- | --- |
| 1. Government of Canada. (2022, 06 14). *Treasury Board of Canada Secretariat.* Retrieved from Backgrounder: [**Government of Canada suspends mandatory vaccination for federal employees**](https://www.canada.ca/en/treasury-board-secretariat/news/2022/06/backgrounder-government-of-canada-suspends-mandatory-vaccination-for-federal-employees.html) | Announcement form the Government of Canada | Yes | N/A | N/A |
| 1. Ssentongo, P., Ssentongo, A.E., Voleti, N. *et al*[*.* **SARS-CoV-2 vaccine effectiveness against infection, symptomatic and severe COVID-19: a systematic review and meta-analysis**](https://doi.org/10.1186/s12879-022-07418-y). *BMC Infect Dis* **22,**439 (2022). | Peer-reviewed systematic review | Yes | None | No |
| 1. Feikin DR, Higdon MM, Abu-Raddad LJ, Andrews N, Araos R, Goldberg Y, Groome MJ, Huppert A, O'Brien KL, Smith PG, Wilder-Smith A, Zeger S, Deloria Knoll M, Patel MK. [**Duration of effectiveness of vaccines against SARS-CoV-2 infection and COVID-19 disease: results of a systematic review and meta-regression**](https://doi.org/10.1016/S0140-6736(22)00152-0). Lancet. 2022 Mar 5;399(10328):924-944. | Peer-reviewed systematic review | Yes | Coalition for Epidemic Preparedness Innovations | Yes  Grade quality appraisal tool: moderate  (The authors believe that the true effect is probably close to the estimated effect) |
| 1. World Health Organization. (2022, May 17). [**Interim statement on the use of additional booster doses of Emergency Use Listed mRNA vaccines against COVID-19.**](https://www.who.int/news/item/17-05-2022-interim-statement-on-the-use-of-additional-booster-doses-of-emergency-use-listed-mrna-vaccines-against-covid-19) | Recommendation by World Health Organization | Yes | WHO | No |
| 1. COVID-END and Coronavirus Variants Rapid Response Network. (2022, June 22). [**COVID-19 Living Evidence Synthesis #6 - What is the efficiency and effectiveness of available COVID-19 vaccines in general and specifically for variants of concern**](https://www.mcmasterforum.org/docs/default-source/product-documents/living-evidence-syntheses/covid-19-living-evidence-synthesis-6.38---what-is-the-efficacy-and-effectiveness-of-available-covid-19-vaccines-in-general-and-specifically-for-variants-of-concern.pdf?sfvrsn=be5dca50_5). | Rapid review and Meta analysis | Yes | Canadian Institutes of Health Research (CIHR) and the Public Health Agency of Canada (PHAC) | Yes  Grade appraisal tool: low certainty evidence  (single RCT or observational study with serious risk of bias or multiple low to serious risk of bias observational studies with inconsistent findings) |

**Table 1.** Demographics/ Participants’ background

|  | **Total**  **N (%)** | **Users**  **N (%)** | **Non-Users**  **N (%)** |
| --- | --- | --- | --- |
| **Total** | **26 (100)** | **12 (46.15)** | **14 (53.85)** |
| **Gender** |  |  |  |
| *Female* | 14 (53.85) | 7 (58.33) | 7 (50) |
| *Male* | 12 (46.15) | 5 (41.67) | 7 (50) |
| *Other* | 0 (0) | 0 (0) | 0 (0) |
| *Did not disclose* | 0 (0) | 0 (0) | 0 (0) |
| **Age** |  |  |  |
| *<30* | 2 (7.69) | 0 (0) | 2 (14.29) |
| *30-39* | 9 (34.62) | 4 (33.33) | 5 (35.71) |
| *40-50* | 7 (26.92) | 3 (25) | 4 (28.57) |
| *>50* | 7 (26.92) | 4 (33.33) | 3 (21.43) |
| **Highest education attained** |  |  |  |
| *Bachelor’s degree* | 2 (7.69) | 2 (16.67) | 0 (0) |
| *Master’s degree* | 11 (42.31) | 3 (25) | 8 (57.14) |
| *PhD* | 5 (19.23) | 4 (33.33) | 1 (7.14) |
| *MD* | 6 (23.08) | 2 (16.67) | 4 (28.57) |
| *Other* | 2 (7.69) | 1 (8.33) | 1 (7.14) |
| **Organization type** |  |  |  |
| *NGO* | 9 (34.62) | 3 (25) | 6 (42.86) |
| *UN specialized agency* | 2 (7.69) | 2 (16.67) | 0 (0) |
| *Government Agency* | 3 (11.54) | 1 (8.33) | 2 (14.29) |
| *Non-profit Humanitarian Organization* | 3 (11.54) | 1 (8.33) | 2 (14.29) |
| *Academic Institution* | 2 (7.69) | 1 (8.33) | 1 (7.14) |
| *Other* | 7 (26.92) | 4 (33.33) | 3 (21.43) |
| **Professional level** |  |  |  |
| *Field worker* | 0 (0) | 0 (0) | 0 (0) |
| *Field manager* | 1 (3.85) | 1 (8.33) | 0 (0) |
| *Program Manager* | 5 (19.23) | 2 (16.67) | 3 (21.43) |
| *Researcher* | 6 (23.08) | 3 (25) | 3 (21.43) |
| *Decision Maker* | 7 (26.92) | 4 (33.33) | 3 (21.43) |
| *Healthcare Provider* | 1 (3.85) | 0 (0) | 1 (7.14) |
| *Advisor* | 5 (19.23) | 2 (16.67) | 3 (21.43) |
| *Other* | 1 (3.85) | 0 (0) | 1 (7.14) |
| **Field/Sector of work** |  |  |  |
| *Cash assistance* | 0 (0) | 0 (0) | 0 (0) |
| *Food security* | 0 (0) | 0 (0) | 0 (0) |
| *Education* | 1 (3.85) | 0 (0) | 1 (7.14) |
| *Livelihoods* | 1 (3.85) | 0 (0) | 1 (7.14) |
| *WASH* | 2 (7.69) | 2 (16.67) | 0 (0) |
| *Protection* | 1 (3.85) | 1 (8.33) | 0 (0) |
| *GBV/SGBV* | 0 (0) | 0 (0) | 0 (0) |
| *Shelter* | 0 (0) | 0 (0) | 0 (0) |
| *Social Stability* | 0 (0) | 0 (0) | 0 (0) |
| *Health* | 16 (61.54) | 7 (58.33) | 9 (64.29) |
| *Other* | 5 (19.23) | 2 (16.67) | 3 (21.43) |
| **Number of years working with the organization** |  |  |  |
| *<1 year* | 2 (7.69) | 1 (8.33) | 1 (7.14) |
| - 1. *years* | 4 (15.38) | 1 (8.33) | 3 (21.43) |
| *3-5 years* | 3 (11.54) | 0 (0) | 3 (21.43) |
| *>5 years* | 17 (65.38) | 10 (83.33) | 7 (50) |
| **Number of published papers** |  |  |  |
| *0* | 8 (30.77) | 2 (16.67) | 6 (42.86) |
| *1-5* | 8 (30.77) | 2 (16.67) | 6 (42.86) |
| *6-10* | 5 (19.23) | 3 (25) | 2 (14.29) |
| *11-15* | 0 (0) | 0 (0) | 0 (0) |
| *>15* | 5 (19.23) | 5 (41.67) | 0 (0) |
| **Main sources of information** |  |  |  |
| *Textbooks* | 0 (0) | 0 (0) | 0 (0) |
| *Colleagues* | 3 (11.54) | 2 (16.67) | 1 (7.14) |
| *Medline searches* | 6 (23.08) | 2 (16.67) | 4 (28.57) |
| *Cochrane Library* | 1 (3.85) | 1 (8.33) | 0 (0) |
| *Evidence aid* | 3 (11.54) | 3 (25) | 0 (0) |
| *BMJ* | 2 (7.69) | 1 (8.33) | 1 (7.14) |
| *Other* | 10 (38.46) | 3 (25) | 7 (50) |
| *None* | 1 (3.85) | 0 (0) | 1 (7.14) |
| **Number of papers read per week** |  |  |  |
| *0* | 2 (7.69) | 1 (8.33) | 1 (7.14) |
| *1-2* | 12 (46.15) | 3 (25) | 9 (64.29) |
| *3-5* | 5 (19.23) | 4 (33.33) | 1 (7.14) |
| *6-10* | 5 (19.23) | 3 (25) | 2 (14.29) |
| *>10* | 2 (7.69) | 1 (8.33) | 1 (7.14) |
| **Familiarity with Evidence Summaries websites** |  |  |  |
| *Never heard of any* | 3 (11.54) | 1 (8.33) | 2 (14.29) |
| *Heard of some* | 19 (73.08) | 7 (58.33) | 12 (85.71) |
| *Very Familiar* | 4 (15.38) | 4 (33.33) | 0 (0) |

**Table 2.** Preferences for Users

|  | **N (%)** |
| --- | --- |
| **First learn about Evidence Summaries websites** |  |
| *From a colleague* | 4 (33.33) |
| *While attending a meeting/conference* | 0 (0) |
| *From research papers* | 3 (25) |
| *Advertisement* | 0 (0) |
| *Other* | 5 (41.67) |
| **Sources most familiar with to access evidence summaries^1^** |  |
| *Evidence Aid* | 7 (58.33) |
| *Campbell Collaboration* | 4 (33.33) |
| *International Initiative for Impact Evaluation (3ie)* | 4 (33.33) |
| *Relief Web* | 7 (58.33) |
| *Cochrane* | 8 (66.67) |
| *Victoria’s Hub for Health Services and Business (health.vic.gov.au)* | 2 (16.67) |
| *Africa Center for Evidence (University of Johannesburg)* | 0 (0) |
| *Other* | 0 (0) |
| **Extent they trust the information on these websites** |  |
| *Do not trust at all* | 0 (0) |
| *Trust a little* | 0 (0) |
| *Trust fairly* | 5 (41.67) |
| *Extremely trust* | 6 (50) |
| *Depends on the source* | 1 (8.33) |
| **Frequency of referring to evidence summaries for updates on COVID-19 during the pandemic (*per month*)** |  |
| *Rarely (less than 2 times)* | 0 (0) |
| *Occasionally (2 to 4 times)* | 7 (58.33) |
| *Frequently (4 to 6 times)* | 2 (16.67) |
| *Very frequently (more than 7 times)* | 3 (25) |
| **Type of information sought in evidence summaries^1^** |  |
| *Basic information about virus composition* | 1 (8.33) |
| *Virus incubation period* | 5 (41.67) |
| *Virus basic signs and symptoms* | 6 (50) |
| *Virus routes of transmission* | 6 (50) |
| *Misconceptions around the virus* | 4 (33.33) |
| *Management of positive cases* | 7 (58.33) |
| *Management of deceased cases* | 3 (25) |
| *Update on vaccine development* | 6 (50) |
| *Direct impact of COVID-19 on health and other outcomes* | 7 (58.33) |
| *Impact of COVID-19 response on other conditions* | 5 (41.67) |
| *Issues to consider for the recovery period after COVID-19* | 6 (50) |
| *System level information* | 6 (50) |
| *Other* | 2 (16.67) |
| **Type of decision implemented after searching evidence summaries^1^** |  |
| *Wanted to implement new SOPs/ or update current SOPs for our field work* | 6 (50) |
| *Wanted to distribute hygiene kits to the communities we work with* | 0 (0) |
| *Wanted to purchase PPEs* | 1 (8.33) |
| *Wanted to decide which kind of intervention should be prioritized during this period* | 9 (75) |
| *Other* | 2 (16.67) |
| **Extent to which the evidence summaries were informative** |  |
| *Not at all informative* | 0 (0) |
| *A little informative* | 2 (16.67) |
| *Somewhat informative* | 8 (66.67) |
| *Very informative* | 2 (16.67) |
| **Extent information in the evidence summaries influenced final decisions** |  |
| *Not at all* | 0 (0) |
| *Very little* | 0 (0) |
| *Somewhat* | 9 (75) |
| *To a great extent* | 3 (25) |
| **Ever based a COVID-19 related decision solely on information from evidence summaries and/or systematic reviews** |  |
| *Yes* | 5 (41.67) |
| *No* | 7 (58.33) |
| **Rating timeliness of COVID-19 evidence summaries availability (“real-time” evidence)** |  |
| *1 (lowest)* | 0 (0) |
| *2* | 5 (41.67) |
| *3* | 4 (33.33) |
| *4* | 3 (25) |
| *5 (highest)* | 0 (0) |
| **Preferred Evidence Summary website** |  |
| *Evidence Aid* | 4 (33.33) |
| *Campbell Collaboration* | 0 (0) |
| *International Initiative for Impact Evaluation (3ie)* | 1 (8.33) |
| *Relief Web* | 2 (16.67) |
| *Cochrane* | 1 (8.33) |
| *Victoria’s Hub for Health Services and Business (health.vic.gov.au)* | 0 (0) |
| *Africa Center for Evidence (University of Johannesburg)* | 0 (0) |
| *Other* | 4 (33.33) |
| **Main challenges faced while using Evidence Summaries websites^1^** |  |
| *Finding the search bar* | 0 (0) |
| *Websites require moving through several pages to reach the desired information* | 6 (50) |
| *Lack of evidence relating to my field of work* | 2 (16.67) |
| *Lack of evidence relating to crisis areas* | 4 (33.33) |
| *Long loading time* | 0 (0) |
| *Some websites’ interface is not user-friendly* | 1 (8.33) |
| *Numerous pop-up advertisements* | 0 (0) |
| *Other* | 2 (16.67) |
| **Preferred presentation to evidence from systematic reviews** |  |
| *Written summaries* | 6 (50) |
| *Figures and charts* | 0 (0) |
| *Presentation* | 2 (16.67) |
| *Other* | 4 (33.33) |
| **Preferred features in the Evidence Summary^1^** |  |
| *Concise summary of the evidence, including benefits, harms and costs, and implementation considerations and recommendations* | 8 (66.67) |
| *Key messages in bullet point format* | 3 (25) |
| *Information about the research methods of the summarized systematic review* | 4 (33.33) |
| *Assessment of the quality of the evidence* | 5 (41.67) |
| *Infographics* | 4 (33.33) |
| *Indirectness assessment* | 1 (8.33) |
| *Date of search strategy (how up to date is the evidence)* | 5 (41.67) |
| *A link that directs you to the full text source* | 6 (50) |
| *Full text pdf option* | 6 (50) |
| *Full-text Citations* | 2 (16.67) |
| *Other* | 1 (8.33) |
| **Suggestions to make the Evidence Summaries websites easier to use/navigate** |  |
| *Include more search filters* | 1 (8.33) |
| *Include more language options* | 0 (0) |
| *Include more topic options* | 3 (25) |
| *Other* | 0 (0) |

^1^ Can select more than one answer (total is not 100%)

**Table 3.** Preferences for Non-Users

|  | **N (%)** |
| --- | --- |
| **Reasons for not using use Evidence Summaries Websites** |  |
| *No specific reason* | 4 (28.57) |
| *Never heard of them before* | 3 (21.43) |
| *I do not usually need such tools for my work* | 1 (7.14) |
| *I find such kind of evidence as “very little informing”* | 0 (0) |
| *I prefer reading the in-depth evidence for a clearer picture* | 2 (14.29) |
| *Other* | 4 (28.57) |
| **Features would like to see in evidence summary websites (if they were to use them)^1^** |  |
| *Have a search bar* | 12 (85.71) |
| *Have filters by topic* | 14 (100) |
| *Have filters by date* | 7 (50) |
| *Have filters by source of evidence* | 4 (28.57) |
| *Provide access to full texts* | 13 (92.86) |
| *Provide different language options* | 3 (21.43) |
| *Provide a subscription option to receive latest updates* | 3 (21.43) |
| *User friendly/easy to follow platform* | 10 (71.43) |
| *I don’t have anything specific* | 0 (0) |
| *Other* | 0 (0) |
| **Features would like to see within an evidence summary (if they were to use them)^1^** |  |
| *Concise summary of the evidence* | 11 (78.57) |
| *Information about the research methods of the summarized research paper* | 9 (64.29) |
| *Assessment of the quality of the evidence* | 8 (57.14) |
| *Infographics* | 8 (57.14) |
| *Risk of bias assessment* | 0 (0) |
| *Indirectness assessment* | 3 (21.43) |
| *Date of search strategy* | 7 (50) |
| *A link that directs you to the full text source* | 13 (92.86) |
| *Full text pdf option* | 6 (42.86) |
| *Implication’s considerations including contextual factors* | 3 (21.43) |
| *Equity related considerations* | 2 (14.29) |
| *Other* | 0 (0) |
| **Features to help them decide which evidence summary website to choose** |  |
| *Based on credibility of website* | 9 (64.29) |
| *Based on the number of years of website’s activity* | 1 (7.14) |
| *Would rely on a colleague’s previous experience* | 3 (21.43) |
| *Other* | 1 (7.14) |
| **Extent they anticipate evidence summaries will have influence on their future decision making** |  |
| *To a great extent* | 4 (28.57) |
| *To a moderate extent* | 8 (57.14) |
| *To a small extent* | 2 (14.29) |
| *Not at all* | 0 (0) |
| **Anticipate using evidence summaries in the future** |  |
| *Yes* | 9 (64.29) |
| *Maybe* | 5 (35.71) |
| *No* | 0 (0) |

^1^ Can select more than one answer (total is not 100%)
